# Essential oils alleviate coccidiosis impact in broiler chickens: a meta-analysis

**DOI:** 10.5713/ab.25.0267

**Published:** 2025-06-10

**Authors:** Ridho Kurniawan Rusli, Melia Afnida Santi, Nuraini, Mustofa Hilmi, Mirzah, Cecep Hidayat, Arif Darmawan, Khairani, Rita Mutia, Mirnawati, Anuraga Jayanegara, Agung Irawan

**Affiliations:** 1 Department of Nutrition and Feed Technology, Faculty of Animal Science, Universitas Andalas, Padang, Indonesia; 2 Animal Feed and Nutrition Modelling (AFENUE) Research Group, Department of Nutrition and Feed Technology, Faculty of Animal Science, IPB University, Bogor, Indonesia; 3 Department of Animal Science, Feed Technology Study Program, Polikteknik Negeri Lampung, Lampung, Indonesia; 4 Study Program of Livestock Product Processing Technology, Politeknik Negeri Banyuwangi, Banyuwangi, Indonesia; 5 Research Center for Animal Husbandry, Research Organization for Agriculture and Food, National Research and Innovation Agency of Indonesia, Bogor, Indonesia; 6 Department of Animal Nutrition and Feed Technology, Faculty of Animal Science, IPB University, Bogor, Indonesia; 7 Graduate School of Animal Science, Faculty of Animal Science, Universitas Andalas, Padang, Indonesia; 8 Vocational Program in Animal Husbandry, Vocational School, Universitas Sebelas Maret, Surakarta, Indonesia

**Keywords:** Alternative Antibiotics, *Eimeria*, Feed Additive, Intestinal Health, Poultry

## Abstract

**Objective:**

This meta-analysis aims to examine the efficacy of essential oils (EO) as an anticoccidial alternative on broiler chickens under coccidia challenged trials, focusing on performance indicators including average daily gain (ADG), feed intake (FI), feed conversion ratio (FCR), final body weight (BW), mortality, and intestinal lesion.

**Methods:**

A random-effects model was performed using the *metafor* package in R software. In a subgroup meta-analysis, treatment groups including coccidia-infected birds [C+], C+ group treated with EO [C+EO] or antibiotics [C+AB], and a non-infected control group treated with EO or AB, were compared against the control group [CON].

**Results:**

As expected, C+ birds had lower (p<0.001) final BW and ADG as well as higher (p<0.001) FCR. Administration of either EO or AB on birds with coccidiosis infection resulted in similar final BW, ADG, FI, and FCR with CON birds, suggesting the comparable effectiveness of EO and AB to alleviate the adverse effects of coccidiosis. Broilers on the C+ group exhibited increased small intestine damage as shown by the greater (p<0.001) lesion score, but the mortality was not different from CON and other treatment groups. The Eimeria oocyst count was lower on birds treated with EO than on the infected birds, with an average suppression of 42.11%. Meta-regression demonstrated that C+ birds had inferior FI and ADG than CON and C+EO birds. However, high heterogeneity between studies was identified in all measured outcomes as shown by *I^2^*>75%, suggesting wide variability among study conditions.

**Conclusion:**

EO may serve as an alternative antibiotic to mitigate the negative impacts of coccidiosis infection in broiler chickens.

## INTRODUCTION

Coccidiosis in broiler chickens is one of the most critical worldwide diseases affecting the poultry industry. Blake et al [1] reported that the global estimate of economic losses due to coccidiosis is around £10.4 billion/year (or equivalent to £0.16/chicken produced). It is an intestinal disease caused by protozoan parasites of the genus Eimeria [2–4], which can cause damage to the intestines, reduce appetite, inhibit growth, and damage the immune system [5]. At least seven to nine species of Eimeria are reported worldwide; these different species can live in various intestinal tracts with different pathogenic rates [3]. Some of the most pathogenic and prevalent species are *Eimeria acervulin*a, *Eimeria maxima*, and *Eimeria tenella* [6]. Due to the high prevalence of coccidiosis in poultry farms, the use of anticoccidial (antibiotics) has been a routine practice for controlling coccidiosis. The high intensity of anticoccidial use may pose risks of antimicrobial resistance [7–9], requiring natural feed additives as an alternative to antibiotics to control the coccidia incidence effectively.

In recent years, studies have been conducted on the developed natural feed additives that are safe to prevent and overcome coccidiosis in broiler chickens. Essential oils (EOs) have gained special attention due to their antioxidant, antimicrobial, and immunomodulatory properties [10–12]. EOs are distilled products from volatile and aromatic plants that have shown a broad spectrum against various pathogenic bacteria in poultry [13,14]. EOs contain terpenoids and aliphatic hydrocarbons [15]. These compounds are effective for improving the performance of broiler chickens [16], intestinal health, and reducing lesions and oocyst count caused by coccidiosis [17]. On the other hand, the addition of EOs in the diets of broiler chickens vaccinated against coccidiosis did not affect the performance [18], lesion, or oocyst count [19].

Although several narrative review articles [2–4,20,21] on coccidiosis have provided excellent fundamental knowledge on this topic, no unbiased quantitative approach, i.e., using meta-analysis has been reported, especially regarding the efficacy of EOs application. Meta-analysis is an effective method for gaining a more comprehensive understanding of certain effects or relationships by combining results from multiple studies conducted across different locations, times, or populations [22]. Therefore, this study aimed to investigate the effects of EOs on broiler chickens challenged with coccidiosis, focusing on performance indicators including average daily gain (ADG), feed intake (FI), feed conversion ratio (FCR), mortality, lesion scores (caecal and small intestine), and oocyst excretion, through meta-analysis study approach.

## MATERIALS AND METHODS

### Articles search and database preparation

This systematic review and meta-analysis methodology followed the PRISMA guidance that involves study selection, data extraction and filtering, and synthesis of relevant information [23]. The main questions of this meta-analysis were “can essential oils replace antibiotic administration on broiler chickens under coccidiosis challenge?” and “to what extent can essential oils alleviate the severity of coccidiosis in broiler chickens?”. Based on these questions, we followed PICO (Population, Intervention, Comparator, and Outcome) items — Population (broiler chickens), Intervention (Eimeria induced coccidiosis condition and EO or antibiotic treatment on the coccidiosis infected condition), Comparator (control treatment without coccidiosis induced condition), and Outcome (final body weight [BW]; FI; ADG; FCR; mortality, and lesion score)—to identify the studies that could be included in the analysis.

**Figure 1:**
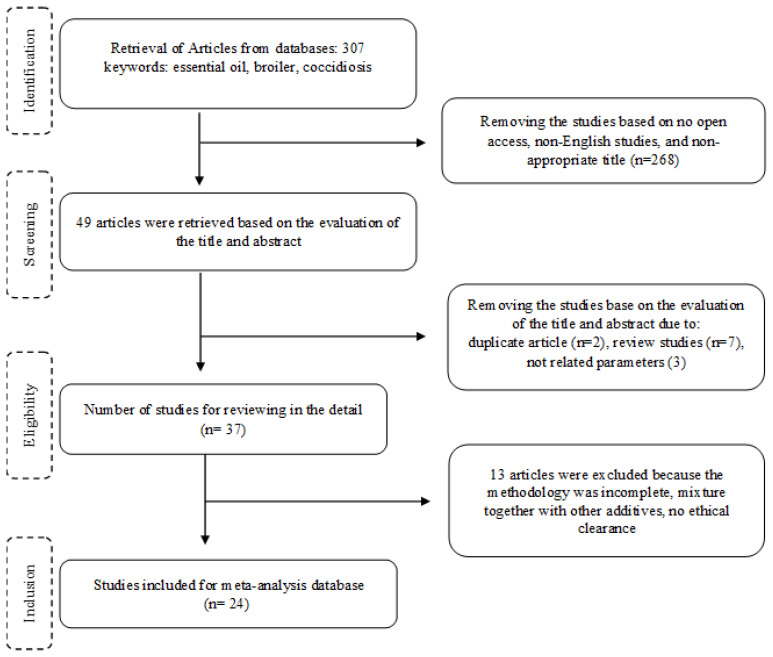
Flow chart of identification and selection process of articles used in meta-analysis.

Articles were searched from scientific electronic databases such as ScienceDirect (https://www.sciencedirect.com/), PubMed (https://pubmed.ncbi.nlm.nih.gov/), Web of Science (https://www.webofscience.com/), and DOAJ (https://doaj.org/) without restriction on publication year. The keywords “essential oil”, “broiler”, and “coccidiosis” were used to assist in the selection of articles; 307 articles were found from all online platforms and then imported into the reference manager (Mendeley version v2.132.0; Mendeley, Elsevier) for selection purposes following PRISMA guidelines [23]. Several inclusion criteria were used to select articles for inclusion in the database: (a) *in vivo* trials on broiler chickens published in English; (b) compared at least three experimental treatments: control, coccidiosis-challenged, and coccidiosis-challenged treated with any type or source of EOs; or exclusively using EOs or antibiotic; (c) feeding only through feed; (d) reported at least one response variable (BW, FI, ADG, FCR, mortality, lesion scores, oocysts excretion) with respective variance (standard deviation [SD] or standard error of the means [SEM]). The SD was calculated from SE using the equation (SD = SE×√n) [24], where n = number of replicates. Several exclusion criteria were also used, including non-peer reviewed article, no ethical clearance was specifically indicated in the article, in vitro or laboratory scale study on Eimeria species, and field surveillance with uncontrolled experimental design, The selection process resulted in 24 final studies that met the eligibility criteria (Figure 1).

### Extraction and description of data

All relevant information from the 24 selected articles was extracted in a dataset with the summary in the Supplement 1, including author name and year of publication, strain, sex, rearing period, type of EOs, dose used, and type of challenge. Articles were obtained from 2003 to 2021. The total number of broiler chickens was 13,396 birds, with the majority of Ross 308 (50%), Cobb 500 (40%), and others (10%) used in this study. The variables included in the database were broiler chickens’ performance (BW gain, feed consumption, feed conversion, and mortality), lesion score (small intestine and cecum), and oocyst excretion. The dose of EOs used was mg/kg feed.

### Assessment of the potential bias of publication

**Figure 2:**
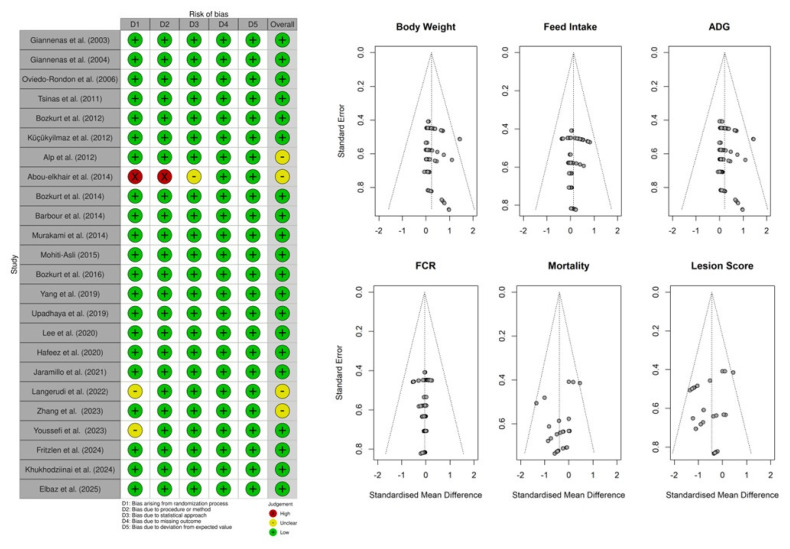
Funnel plot of some major parameters to assess the publication bias. ADG, average daily gain; FCR, feed conversion ratio.

The study’s limitations, commonly referred to as risks of bias from each study, were assessed using the Cochrane Collaboration framework [25], funnel plot, and Egger’s test. This evaluation was based on several criteria: (a) randomization and animal handling; (b) procedure and outcomes’ measurements; (c) statistical approach; (d) result variability; and (e) reported outcomes and the variance. Two independent members assessed these criteria and were validated by another member following a hierarchical rating system. The rating system was as follows: A “low risk” rating received a score of 3, “some concerns” a score of 2, and “high risk” a score of 1. The overall risk of bias was determined by calculating the total score, with studies scoring ≤7 excluded from the analysis [26]. A summary table of individual study assessments was uploaded to the Robvis (Risk-of-bias VISualization) platform to generate traffic light plots and weighted bar plots [27] with the risk of bias results presented in Figure 2. In addition, funnel plots and Egger’s test were also reported to assess publication bias from the data variability. A sensitivity analysis was performed to assess the stability of treatment effects by influential studies contributing to high heterogeneity or outliers in the measured outcomes. To achieve this, a leave-one-out analysis was conducted [25].

### Meta-analysis

Data were organized in the columns representing the variables and lines (rows) that listed the studies and data points. The number of replicates, the mean value of the control, and the mean value of the comparator (treatment), along with their SD values, were set for each comparison. The negative control group (CON) serves as the control while the other treatments that were coded specifically (C+ = birds infected with coccidia; C+EO = C+ birds treated with EO; C+AB = C+ birds treated with antibiotics; EO = CON birds fed EO-supplemented diet; and AB = CON birds fed antibiotics containing diet) served as the comparators. Other potential moderating variables were also arranged in the dataset, including the country of origin where the trial was conducted, strain of broilers, sex of broilers, type of EO, type of AB, and the administration levels.

Then, the meta-analysis was conducted using the *metafor* package in R [28] using both univariate and multivariate meta-analysis functions. Random effect models were employed, considering the individual trial as a random factor, as they contributed to different study variances. The effect size was calculated following the previously described method [29]. The output was presented as standardized mean differences (SMD) along with their 95% confidence intervals (CI) and was displayed in the forest plots. Between-study heterogeneity was assessed using Cochran’s Q statistic and the *I*^2^ statistic, with the DerSimonian-Laird estimator applied to calculate I^2^ for univariate meta-analysis.

In contrast, the multivariate meta-analysis used maximum likelihood for the estimator. The data on oocyst count were analyzed separately due to the variability reported in each study. Therefore, we opted to transform the oocyst count from birds treated with EOs as a relative suppression rate against those challenged with *Eimeria* oocysts.

Additionally, meta-regression analysis was performed to study and predict each treatment group’s relationship between FI and ADG data using linear mixed model (LMM). The LMMs were carried out using *the lme4* and *lmerTest* packages in R, as explained in previous publications [30,31], and the SE from each study was used to calculate the inverse variance matrix as the weighting factor. Potential covariates were considered in the meta-regression analysis, including the country of origin, bird’s strains, sources of EO, and levels of EO. However, small number of sample size of each category or group limits the exploratory studies which often led to consistently high heterogeneity value. Therefore, we opted to exclude the results of covariates effect.

## RESULTS

### Study characteristics

A total of 24 studies conducted in 11 countries (Brazil, Canada, China, Egypt, Greece, Korea, Iran, Pakistan, Lebanon, Turkiye, and the USA) around the world were collected. The EOs used in this study were derived from various plants and administered at 0–2,000 mg/kg doses through feed. Descriptive statistics showed that the variability in the mean values of the variables of interest was due to the experimental setup, broiler strains, and rearing period. As shown in Supplement 1, majority of the experiments were conducted between 35 days (37.5%) and 42 days (54.2%) of rearing period and only 1 study in which the birds were reared on 28 and 49 days. In all experiments, authors indicated that the birds were reared under environmentally controlled rooms to adhere to the animal welfare and management guideline standard of the broiler strain. The broilers’ strains were relatively homogenous, with Ross and Cobb representing 33.3% and 58.3% of the strains reported in the dataset, respectively. Therefore, although the results might be influenced by strains, rearing period, and management, the effect is assumed to be minimum to influence the experimental treatments. In addition, the data in each study were checked for acceptability according to the standard of performance objective for each strain established by the breeding company. Assessment of risks of bias from publication shows a minimum bias of publication as shown in the traffic light plot (Figure 2). Funnel analysis also reveals low bias from the dataset, with standard errors clustering inside the triangle and no strong asymmetry between positive and negative effects (Figure 2).

### Production and growth performance

**Figure 3:**
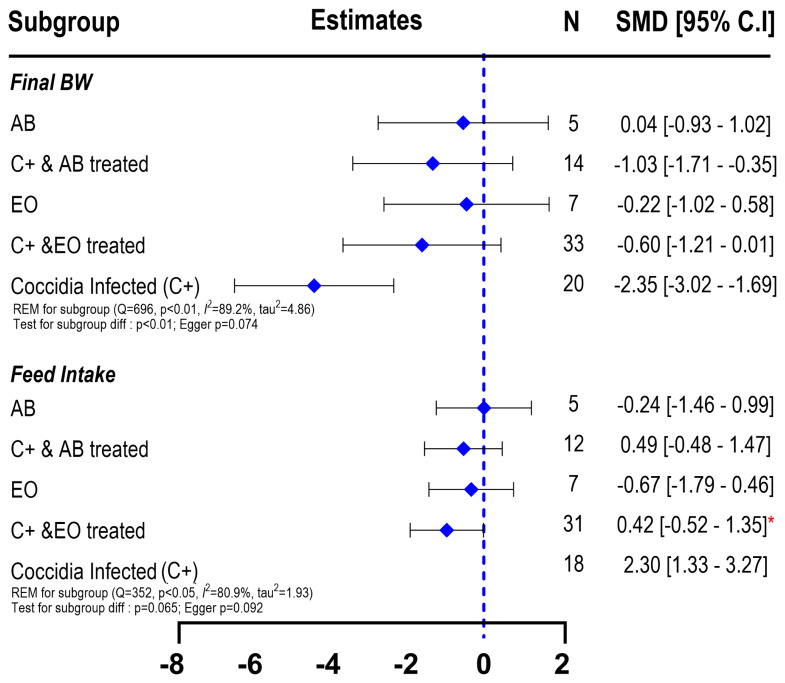
Forest plot of subgroup meta-analysis showing the effects of essential oils as alternative antibiotics on final body weight and feed intake of broiler chickens under coccidiosis-challenged studies. The x-axis shows the SMD; the central-blue line represents the zero effect (SMD = 0) of interventions; the diamond-blue symbol represents the SMD (subgroup effect). Asterisk symbol is provided (* p<0.05; ** p<0.01; *** p<0.001) in each subgroup when there is significant effect. N is the sample size. BW, body weight; SMD, standardized mean differences; EO, essential oil.

The multivariate subgroup meta-analysis for final BW is presented in Figure 3. Broiler chickens infected with coccidiosis conditions (C+ group) exhibited a substantial reduction in their final BW (SMD −4.35; 95% CI: −6.39 to −2.31). Although a notably lower than CON, EOs administration on infected birds (C+EO) resulted in statistically not significant effect (SMD = −1.58; 95% CI: −3.61 to 0.44; p>0.05) compared to the CON group. Similarly, antibiotics administration also resulted in similar final BW (C+AB; SMD = −1.31; 95% CI: −3.36 to 0.74; p>0.05) with the CON birds, indicating that EO and AB comparably showed recovery effect on infected birds. Unexpectedly, EO (SMD = −0.44; 95% CI: −2.56 to 1.67; p<0.05) and AB (SMD = −0.53; 95% CI: −2.71 to 1.65; p<0.05) did not show an improved effect on the final BW of the birds when given to non-infected birds. High heterogeneity (QE = 698; I^2^ = 89%) was identified with significant variability (τ^2^ = 4.86, p<0.001).

**Figure 4:**
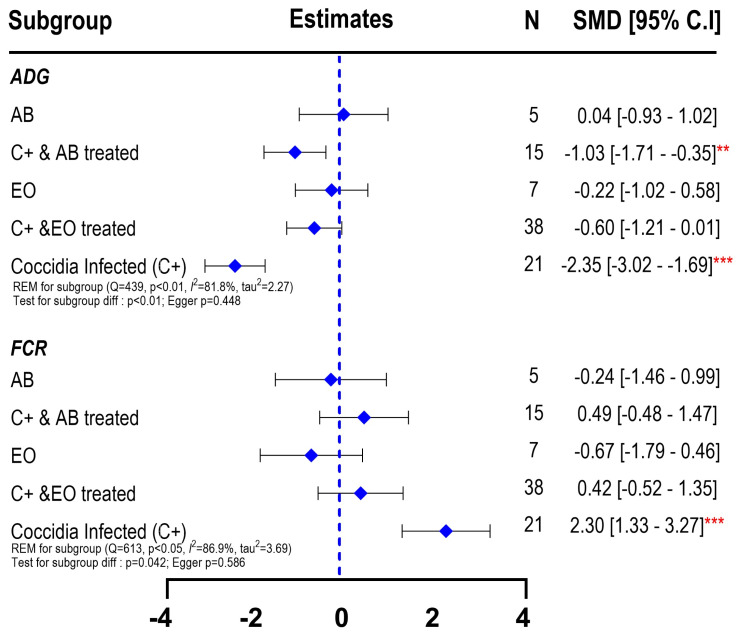
Forest plot of subgroup meta-analysis showing the effects of essential oils as alternative antibiotics on average daily gain, daily feed intake, and feed conversion ratio of broiler chickens under coccidiosis-challenged studies. The x-axis shows the SMD; the central-blue line represents the zero effect (SMD = 0) of dietary interventions; the diamond-blue symbol represents the SMD (subgroup effect). Asterisk symbol is provided (* p<0.05; ** p<0.01; *** p<0.001) in each subgroup when there is significant effect. N is the sample size. SMD, standardized mean differences; ADG, average daily gain; EO, essential oil; FCR, feed conversion ratio.

Similar to the results of final BW, the subgroup meta-analysis for ADG showed that coccidia-infected broilers (C+) had substantial lower ADG (SMD = −2.35; 95% CI: −3.02 to −1.69) than birds in CON group. The EO treatment on birds with coccidia infection (C+ EO) slightly recovered the ADG (SMD = −0.60; 95% CI: −1.21 to 0.01) in comparison to the C+ birds (Figure 4). Despite a degree of recovery being observed, AB treatment to coccidia-infected chickens still resulted in lower ADG (SMD = −1.03; 95% CI: −1.71 to −0.35) than CON birds, suggesting a limited effect of antibiotics in mitigating coccidiosis-induced growth suppression. Administration of AB (SMD = 0.04; 95% CI: −0.93 to 1.02) or EO (SMD = 0.22; 95% CI: −1.02 to 0.58) also had a lack of effect to improve ADG of CON birds (p>0.05).

The forest plot of subgroup meta-analysis on FI (Figure 4) showed only a numerical reduction of FI in birds infected with coccidiosis (SMD = −0.83; 95% CI = −1.79 to 0.14). The C+ birds treated with AB or CON birds administered with AB or EO had similar FI with CON birds (p>0.05). A lower FI was observed on C+EO birds (SMD = −0.95; 95% CI = −1.89 to −0.01). High heterogeneity was also observed with between-studies significant variability (I^2^ = 80.9%; τ^2^ = 1.93, p<0.001). The test for subgroup differences yielded a borderline significance (p = 0.065), indicating variability in treatment responses. The Egger’s test for publication bias yielded a p-value of 0.092, suggesting no substantial publication bias in the included studies. Overall, these results indicate that EOs may partially alleviate the effects of coccidiosis on growth performance and FI but require further investigation to optimize their efficacy.

The forest plot of subgroup analysis of FCR demonstrated that coccidia-infected birds (C+) exhibited a significantly higher FCR (SMD = 2.30; 95% CI: 1.33 to 3.27) (Figure 4), suggesting low feed efficiency when birds are infected with coccidiosis. Treating C+ birds with EO resulted in a moderate improvement in FCR (SMD = 0.42; 95% CI: −0.52 to 1.35). However, it did not completely restore feed efficiency as it showed no significant difference with CON birds. AB treatment also demonstrated similar effects (SMD = 0.49; 95% CI: −0.48 to 1.47). When administered to the CON group without coccidia infection, the FCR was not different from the CON group, likely due to high between-studies heterogeneity (I^2^ = 86.9%; τ^2^ = 3.93, p<0.001).

**Figure 5:**
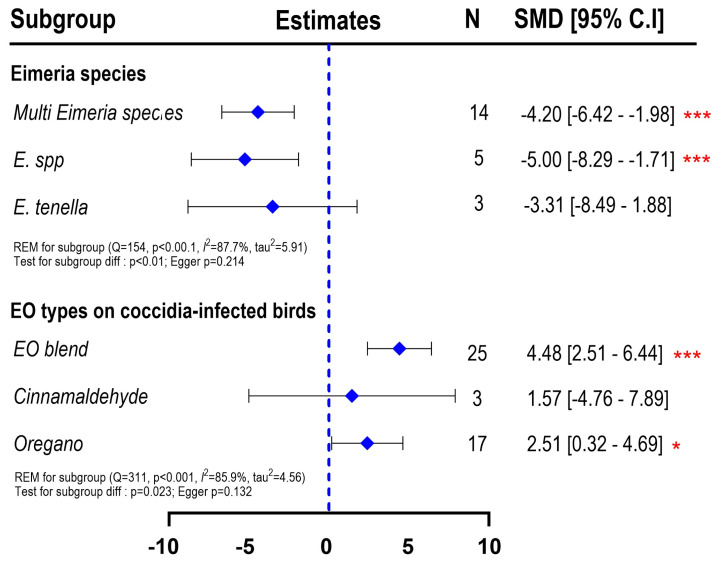
Forest plot of subgroup meta-analysis to explain the effects of different Eimeria species for coccidia challenge on final BW of broiler chickens and the effects of different types of EO on broiler chickens that received coccidia challenge. The x-axis shows the SMD; the central-blue line represents the zero effect (SMD = 0) of dietary interventions; the diamond-blue symbol represents the SMD (subgroup effect). Asterisk symbol is provided (* p<0.05; ** p<0.01; *** p<0.001) in each subgroup when there is significant effect. N is the sample size. SMD, standardized mean differences; EO, essential oil; BW, body weight.

Figure 5 provides empirical evidence that the deleterious effects among Eimeria species to induce coccidiosis were different (p<0.01). Specifically, broiler chickens infected with multispecies of Eimeria (*E. tenella, E.* spp., *E. maxima*) or *E.* spp. resulted in significantly lower final BW (p<0.001) while infection with E*. tenella* alone showed numerically lower BW but no difference with Cthe ON group (SMD = −3.31; 95% CI = −8.49 to 1.88; p>0.05). Different efficacy was also found on the types of EO whereas EO blend commonly sourced from commercial products (SMD = 4.48; 95% CI = 2.51 to 6.44; p<0.001) and oregano-based EO (SMD = 2.51; 95% CI = 0.32 to 4.69; p<0.05) resulted in improved BW while cinnamaldehyde-based EO did not improve BW of broiler chickens infected with coccidia.

### Mortality, intestinal lesion, and oocyst suppression rate

**Figure 6:**
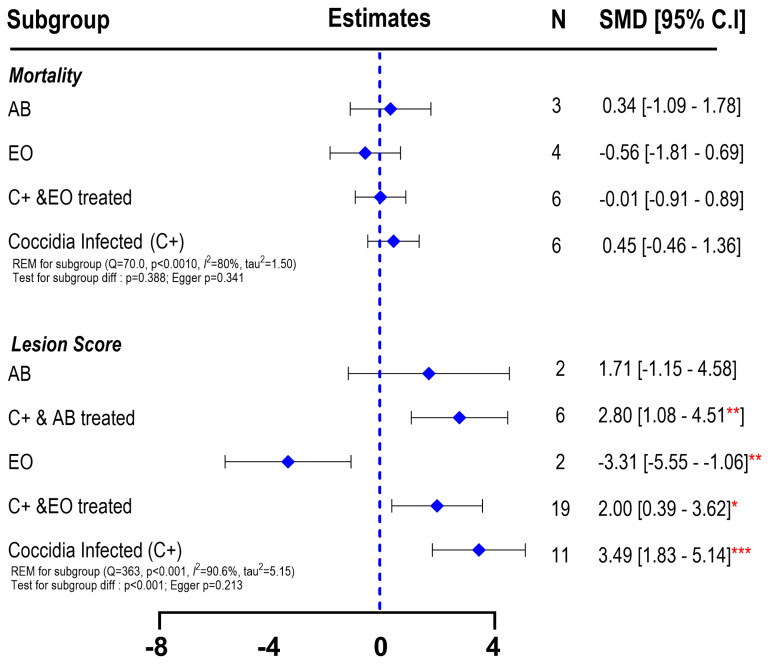
Forest plot of subgroup meta-analysis showing the effects of essential oils as alternative antibiotics on mortality and lesion score of broiler chickens under coccidiosis-challenged studies. The x-axis shows the SMD; the central-blue line represents the zero effect (SMD = 0) of dietary interventions; the diamond-blue symbol represents the SMD (subgroup effect). Asterisk symbol is provided (* p<0.05; ** p<0.01; *** p<0.001) in each subgroup when there is significant effect. N is the sample size. SMD, standardized mean differences; EO, essential oil.

The results of subgroup meta-analysis on mortality rate showed overall no statistically significant effects. Coccidia-infected birds (C+) had a numerically higher mortality rate than other groups. At the same time, treatment with EO slightly reduced the mortality rate, but the effect was only numerical (Figure 6).

The intestinal lesion score is an indicator of intestinal damage due to the coccidiosis infection. The results of subgroup meta-analysis clearly showed that coccidia infections resulted in a greater lesion score (3.49; 95% CI: 1.83 to 5.14; p<0.001) compared to the CON group, indicating the severe effect on intestinal damage caused by coccidiosis. Treatment with AB or EO did not result in a recovery effect, as shown by the higher lesion score on those groups compared to CON (Figure 6). As expected, EO had a lower lesion score when fed to the birds without coccidia infections (CON) (SMD = −3.31; 95% CI = −5.55 to −1.06), although the number of studies was low (n=2). The heterogeneity was high for mortality (I^2^ = 80%) and lesion score (I^2^ = 90.6%). Antibiotic treatment showed no difference in lesion score with the CON birds. Egger’s test for publication bias yielded a p-value of 0.341 for mortality and 0.213 for lesion scores, suggesting minimal publication bias.

**Figure 7:**
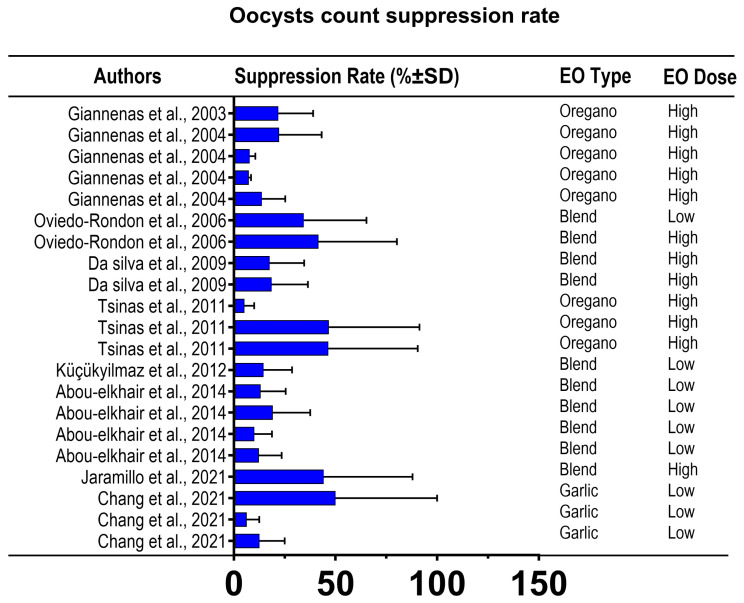
Suppression rate of Eimeria oocysts count from *in vivo* studies evaluating the efficacy of essential oils on broiler chickens challenged with Eimeria oocysts infection. SD, standard deviation; EO, essential oil.

The suppression or sporulation rate of *Eimeria* oocysts counts from 10 *in vivo* studies from broiler chickens that were treated with EOs is shown in Figure 6. Administration with EOs resulted in a high variability of reduction percentage on oocyst counts, ranging from 0.27% to 100% compared to non-treated chickens, with an average of 42.11%. Importantly, a low suppression rate (<20%) indicating low efficacy of EOs in suppressing *Eimeria* oocysts was only found in 24% of the dataset, while the rest showed moderate to high suppression rates (Figure 7).

### Relationships between feed intake and average daily gain

**Figure 8:**
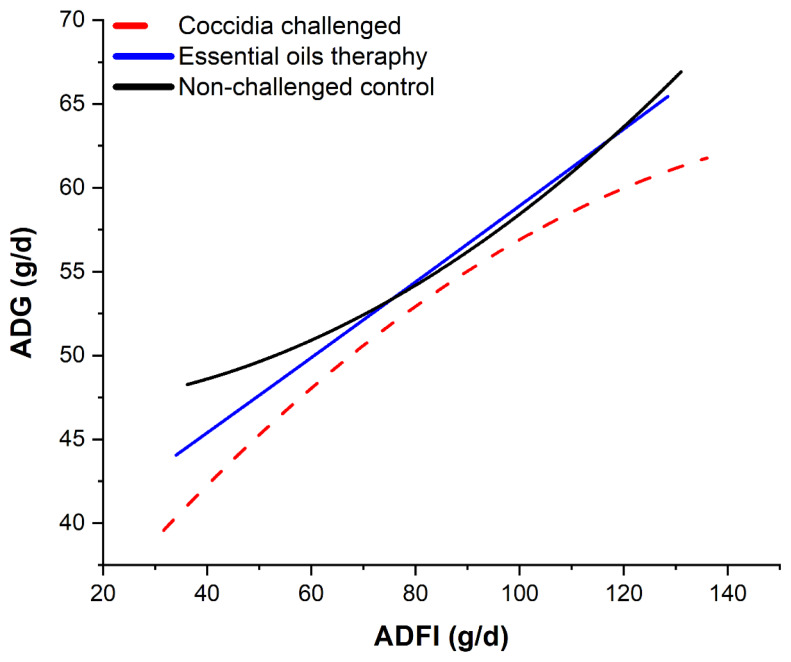
The relationship between the variance of ADFI (g/d) with ADG (g/d) of broiler chickens as the implication of coccidia challenged trials and essential oils therapeutic effects. Polynomial regression equations of ADG as a function of ADFI for each situation were: y = −0.0011x^2^+0.402x+27.99; R^2^ = 0.87; RSE = 4.64; p = 0.009 (coccidia challenged birds); y = 0.0012x^2^−0.0054x+46.88; R^2^ = 0.93; RSE = 4.71; p = 0.003 (control or non-coccidia-challenged); and y = 4E−05x^2^+0.220x+36.53; R^2^ = 0.95; RSE = 4.72; p = 0.003 (EOs therapy). ADG, average daily gain; ADFI, average daily feed intake; RSE, relative standard error.

Figure 8 shows the predicted ADG as a function of FI from three major conditions: normal conditions (CON group), coccidia-infected condition, and EO treatment against coccidia infection. Overall, curvilinear functions were observed in the relationship of FI and ADG, as expected. Birds infected with coccidia demonstrated the lowest ADG at constant or similar FI, while EO administration on infected birds or during a coccidia challenge exhibited similar ADG with the CON group, indicating the potential of EO to alleviate the detrimental effects of coccidiosis, although larger dataset is needed for more robust research synthesis of the role of EO on coccidiosis infection in broiler chickens.

## DISCUSSION

### Essential oils could mitigate growth performance impairment from coccidiosis infection

The significant economic losses due to coccidia infection in broiler chickens have prompted scientists to examine the suitability of natural-based feed additives to ameliorate the detrimental effects on mortality, morbidity, growth, and overall health. Interest in the exploitation of EOs as anticoccidial is based on their strong and wide-spectrum antimicrobial properties as well as other multiple benefits such as anti-inflammatory and antioxidant [32,33]. This meta-analysis aimed to provide quantitative evidence on whether EO could serve as an alternative for broilers under coccidiosis infections.

Overall, the results on final BW and ADG indicate a substantial reduction of final BW due to Eimeria infection, highlighting the detrimental impact of coccidiosis on growth performance. The suppression caused by coccidiosis is attributed to intestinal damage, immune system impairment, and nutrient absorption [9,32,34]. Eimeria species invade epithelium, destroy the enterocytes, and thus disrupt overall gut integrity. It has been widely reported that Eimeria infections induce the release of pro-inflammatory cytokines, leading to various degrees of inflammation [35]. The intestinal damage and inflammation are two primary conditions that cause poor nutrient absorption and thus limit the nutrient availability for broiler growth. In addition, the infected birds may use absorbed nutrients to maintain their basic physiological functions because broilers that experienced microbial infections require a higher energy demand for their growth. Thus, lower ADG with similar Fi is translated into lower final BW and poor FCR.

Feed consumption is a significant factor in influencing broiler chickens’ performance, where decreased feed consumption has a negative impact on BW gain and final BW. In this study, coccidiosis challenge reduced feed consumption and BW gain. Feed consumption of broiler chickens with coccidiosis challenge was lower than the EO treatment and the EO additive. Coccidiosis reduces daily feed consumption [36]. The decrease in feed consumption is due to the production of inflammatory cytokines that provide negative feedback to the central nervous system, thus reducing the animal’s appetite [37]. Coccidiosis reduced feed consumption in broiler chickens due to Eimeria parasite infection, causing severe damage to the gastrointestinal tract, particularly the intestinal mucosa, which disrupts the normal function of digestion and nutrient absorption [38]. When chickens were infected, the parasite damaged the intestinal epithelial lining through its life cycle, including replication and release of merozoites, which caused inflammation, bleeding, and ulceration [39]. These conditions lead to discomfort in the chickens, resulting in reduced appetite. In addition, the immune response activated by the chicken’s body to fight the infection produced proinflammatory cytokines, such as interleukin-1 (IL-1) and tumor necrosis factor-alpha (TNF-α), which were known to suppress appetite regulation [40].

This meta-analysis study showed that coccidia challenge had a significant effect on increasing the FCR. In the study, the value of broiler chickens FI decreased as well as BW gain so that the FCR increased. Coccidiosis challenge in broilers chickens significantly affects performance values, but the provision of EOs has also not improved performance in broilers chickens. The severity of coccidiosis is classified into three levels [41]: mild coccidiosis (no side effects); subclinical coccidiosis; and clinical coccidiosis. Subclinical coccidiosis can result in reduced growth, reduced FI (which can lead to anorexia), reduced feed digestibility, and reduced feed efficiency [42].

The amelioration effect of EO on broiler chickens infected with coccidiosis differed depending on the type of EO. Commercial EO products that often-used multiple types of EO were found to be more effective than oregano and cinnamaldehyde to be used to alleviate coccidiosis. The difference in efficacy could be attributed to different anticoccidial activity and potential synergistic roles of different EO when formulated as a mixture or blend. A comparative study of ten EO sources against Eimeria oocyst found only two EO sources rich in chavicol showed the strong efficacy against Eimeria oocyst [43]. Other studies study also demonstrated that EO blend (eugenol, thymol, piperine) or a mixture of cinnamaldehyde and citral EO showed greater efficacy than when cinnamaldehyde or citral were administered alone [44,45].

### Essential oils alleviate intestinal damage due to coccidiosis

The higher lesion score of the small intestine confirmed the higher intestinal damage of infected birds in this study. Coccidian challenge can reduce digestive enzymes [46], reduce intestinal villi length, and disrupt intestinal morphology [47]. EOs have antioxidant and anti-inflammatory activities, thus protecting intestinal epithelial cells from oxidative damage [48,49]. EOs exert positive effects by repairing damage from intestinal lesions through their bioactive properties. Eugenol in EOs has antimicrobial activities that directly suppress the growth and life cycle of Eimeria parasites [35,50], thereby reducing further damage to the intestinal mucosa. In addition, anti-inflammatory properties of EOs inhibit the release of pro-inflammatory cytokines, hence reducing the level of inflammation and accelerating the tissue recovery process [35]. The potent antioxidant activity of EOs also contributed to neutralizing free radicals generated during oxidative stress and protected epithelial cells from further damage [49].

By contrast, the supplementation of EOs to the CON birds increased ADG, confirming previous meta-analysis studying the effects of EO as a growth-promoting additive [16]. It is known that EO can improve the digestive system by maintaining microbial balance and increasing nutrient absorption [51], as well as stimulate the activity of digestive enzymes in the small intestine and pancreas [52]. The EOs exert their anticoccidial effects, including modulation of gut microbiota, enhancement of intestinal barrier function, and stimulation of the host’s immune response [53,54]. Additionally, EOs have been reported to reduce oxidative stress and inflammation in the gut, which are commonly associated with coccidia infections [50,55]. These attributes make EOs a compelling alternative to synthetic anticoccidial drugs, aligning with the global trend toward sustainable and antibiotic-free poultry production.

Regarding the antimicrobial properties, EOs are effective against Eimeria parasites that cause coccidiosis. By suppressing parasite replication, EOs reduce damage to the intestinal epithelium that often occurs due to coccidiosis infection [35]. Moreover, the anti-inflammatory and antioxidant properties of EOs help reduce inflammation and oxidative stress induced by the infection, thereby accelerating the recovery of intestinal tissues [55]. EOs also play a role in improving the balance of gut microflora by inhibiting pathogenic microorganisms and supporting the growth of beneficial bacteria, such as Lactobacillus [35]. This increases the efficiency of digestion and nutrient absorption. In addition, EOs can stimulate the secretion of digestive enzymes, such as amylase and protease [56], further improving feed utilization. Despite their promising benefits, the effectiveness of EOs in controlling coccidiosis varies depending on their composition, dosage, and mode of administration, which requires further investigation, including how EOs may synergistically work with other natural feed additives.

### Essential oils effectively sporulate Eimeria oocysts

Oocyst excreta is one of the essential parameters when studying coccidiosis infection in broiler chickens because broilers exposed to coccidiosis excreta contain coccidian oocysts. Coccidian infection increases the number of oocysts per gram of excreta [57]. Coccidiosis infection occurs when chickens eat oocysts that circulate in the environment, which then invade and damage mucosal cells in the intestine [44]. Feed containing EOs can reduce the number of excreta oocyst. EO additive is one of the alternative conventional anticoccidials in poultry feed [58]. EOs contain high antioxidants and active immunomodulators. EOs can directly kill Eimeria oocysts and stop sporulation by penetrating the Eimeria oocyst wall.

EOs are widely used to control coccidiosis [59], but with varying degrees of efficacy, especially in suppressing oocyst count. Under in vitro experiments, numerous EOs exhibited a powerful destructive effect on Eimeria oocysts, i.e., oocysts treated with EO resulted in the presence of intracellular content (debris) release as a proof of oocysts deforming condition [33]. Similarly, more recent studies demonstrated the high sporulation or inhibition effects on *E. tenella* oocysts and mixed Eimeria species from broilers after D-limonene and citrus sinensis EO treatment [55]. Naturally, EOs are highly lipophilic compounds that could effectively penetrate the cell walls of oocysts and disrupt the intracellular environment of oocysts. The disruption of the outer permeable membrane of Eimeria caused an increase in cytoplasmic membrane permeability. This condition releases lipopolysaccharides, proteins, cellular fatty acids, and leakage, allowing the hydrophobic nature of EOs to penetrate the cell membrane, disrupting the cellular metabolism of the oocyst, leading to cell death [53,60]. Comparative studies are scarce to examine the efficacy of various EOs or EOs’ bioactive compounds. Therefore, it remains inconclusive whether specific active compounds of EOs may be superior to others, as most studies used a mixture of EOs. However, the direct suppressive effect was confirmed in several *in vivo* studies involving broiler chickens [6,17,54]. Given this strong evidence on the efficacy of EOs as anticoccidial agents, increasing numbers of publications further pursued on-farm efficacy as an alternative to coccidiostat (antibiotics).

## CONCLUSION

This meta-analysis provides cumulative evidence on the impairment effects of coccidiosis on the growth and production performance of broiler chickens. The use of EOs blend, regardless of dose, as a feed additive appeared to be effective to alleviate the detrimental effects of coccidiosis. However, the recovery effect of EOs is numerically lower compared to antibiotics or coccidiostat used to control coccidia infections in broiler chickens. This evidence underscores the beneficial application of EOs to be safely used to ameliorate the coccidiosis infection, reducing the potential risk of antimicrobial resistance from the overuse of antibiotics.

## SUPPLEMENTARY MATERIAL

**Supplement 1.** Lists of studies included in the meta-analysis.

**Supplement 2.** Descriptive statistical summary of the dataset.
