# Supplementary material for: Essential oils alleviate coccidiosis impact in broiler chickens: a meta-analysis
Source: Anim Biosci. 2025 Jun 10;38(12):2726–40. doi: 10.5713/ab.25.0267 (PMC12580784; doi:10.5713/ab.25.0267)
Supplement: Supplementary file 1 [file ab-25-0267-Supplementary-1.pdf]

Supplement 1. Lists of studies included in the meta-analysis

Study	Authors	Country	Strain	Sex	Age, d	N birds	Essential oils	Coccidia-challenged method
1	Giannenas et al. [1]	Greece	Cobb 500	Mix	35	90	Oregano EOs	*E. tenella*
2	Giannenas et al. [2]	Greece	Cobb 500	Mix	35	180	Oregano EOs	*E. tenella*
3	Oviedo-Rondón et al. [3]	USA	Cobb 500	Male	49	144	Thymol, eugenol, curcumin, piperine	cocci-vaccinated
4	Tsinas et al. [4]	Greece	Cobb 500	Male	35	300	Oregano EOs	*E. acervulina and E. maxima*
5	Bozkurt et al. [5]	Turkey	Ross 308	Mix	42	832	Oregano, laurel, and lavender	*E. spp*
6	Küçükyilmaz et al. [6]	Turkey	Ross 308	Mix	42	312	Carvacrol, thymol based	*E. spp*
7	Alp et al. [7]	Turkey	Ross 308	N/A	42	1200	Oregano EOs	cocci-vaccinated
8	Gaafar et al. [8]	Egypt	Cobb 500	N/A	42	180	Oregano, thymol, and garlic	*E. spp*
9	Bozkurt et al. [9]	Turkey	Ross 308	Mix	42	400	Oregano, laurel, and lavender	*E. spp*
10	Barbour et al. [10]	Lebanon	Ross 308	N/A	35	160	Cineol, limonene, L-menthol, phellandrene	*E. spp.*
11	Murakami et al. [11]	Brazil	Cobb-500	Male	42	3000	Cashew nut EOs	*E. acervulina, E. maxima, and E. tenella*
12	Mohiti-Asli & Ghanaatparast-Rashti [12]	Iran	Ross 308	N/A	42	150	Oregano EOs	*E. acervulina, E. maxima, and E. tenella*
13	Bozkurt et al. [13]	Turkey	Ross 308	Mix	42	648	Oregano EOs	*E. spp*
14	Yang et al. [14]	Canada	Cobb 500	Male	28	3200	Cinnamaldehyde and citrus	cocci-vaccinated
15	Upadhaya et al. [15]	Korea	Ross 308	Male	35	800	Thymol, eugenol and piperine	cocci-vaccinated
16	Lee et al. [16]	Korea	Ross 308	Male	35	480	Carvacrol, thymol based	cocci-vaccinated
17	Hafeez et al. [17]	Pakistan	Hubbard	Female	42	560	Coconut oil EOs	cocci-vaccinated
18	Jaramillo et al. [18]	Korea	Ross 308	Male	35	210	Oregano and citrus	cocci-vaccinated

19	Langerudi et al. [19]	Iran	Ross 308	Male	42	75	*Psidium guajava*	*E. acervulina, E. maxima, E. necatrix, E. tenella*
20	Zhang et al. [20]	China	Arbor Acres	Male	42	576	Oregano and Clove	*E. acervulina, E. maxima, and E. tenella*
21	Youssefi et al. [21]	Iran	Ross 308	Male	35	105	Oregano and Clove	*E. acervulina, E. maxima, E. necatrix, E. tenella*
22	Fritzlen et al. [22]	USA	Ross 708	Male	42	1152	Garlic and cinnamon	cocci-vaccinated
23	Khukhodziinai et al. [23]	India	Cobb-500	N/A	42	252	Oregano EOs	*E. acervulina, E. maxima, and E. tenella*
24	Elbaz et al. [24]	Egypt	Ross 308	Male	35	600	Oregano EOs	*E. acervulina, E. maxima, and E. tenella*
