# Supplementary material for: Essential oils alleviate coccidiosis impact in broiler chickens: a meta-analysis
Source: Anim Biosci. 2025 Jun 10;38(12):2726–40. doi: 10.5713/ab.25.0267 (PMC12580784; doi:10.5713/ab.25.0267)
Supplement: Supplementary file 2 [file ab-25-0267-Supplementary-2.pdf]

Supplement 2. Descriptive statistical summary of the dataset

Parameters	Unit	Mean	SD	Min	Max
Average daily gain	g/bird/day	63.98	27.40	27.63	144.80
Average daily feed intake	g/bird/day	97.61	26.19	31.60	136.00
Feed conversion ratio	No unit	1.75	0.21	1.36	2.58
Mortality	(%)	3.42	3.20	0.00	10.31
Survival Rate	(%)	96.90	1.10	80.00	100.00
Lesion scores (Cecum)	Arbitrary	4.86	9.64	0.00	35.00
Lesion scores (Small Intestine)	Arbitrary	0.90	1.24	0.00	4.30
Oocysts excretion	(Log^{10}/g)	3.50	1.41	0.00	44.58

REFERENCES

1. Giannenas I, Florou-Paneri P, Papazahariadou M, Christaki E, Botsoglou NA, Spais AB. Effect of dietary supplementation with oregano essential oil on performance of broilers after experimental infection with Eimeria tenella. Arch Anim Nutr. 2003;57(2):99–106.
2. Giannenas IA, Florou-Paneri P, Papazahariadou M, Botsoglou NA, Christaki E, Spais AB. Effect of diet supplementation with ground oregano on performance of broiler chickens challenged with Eimeria tenella Einfluss des Zusatzes von gemahlenem Oregano zur Futterration auf die Leistung von mit Eimeria tenella belasteten Broilerküken. Vol. 68. 2004.
3. Oviedo-Rondón EO, Clemente-Hernández S, Salvador F, Williams P, Losa R. Essential oils on mixed coccidia vaccination and infection in broilers. Int J Poult Sci. 2006;5(8):723–30.
4. Tsinas A, Giannenas I, Voidarou C, Tzora A, Skoufos J. Effects of an oregano based dietary supplement on performance of broiler chickens experimentally infected with Eimeria acervulina and Eimeria maxima. Journal of Poultry Science. 2011;48(3):194–200.
5. Bozkurt M, Selek N, Küçükyilmaz K, Eren H, Güven E, Çatli AU, Çinar M. Effects of dietary supplementation with a herbal extract on the performance of broilers infected with a mixture of Eimeria species. Br Poult Sci. 2012 Jun;53(3):325–32.
6. Küçükyilmaz K, Bozkurt M, Selek N, Güven E, Eren H, Atasever A, Bintaş E, Çatli AU, Çinar M. Effects of vaccination against coccidiosis, with and without a specific herbal essential oil blend, on performance, oocyst excretion and serum IBD titers of broilers reared on litter. Ital J Anim Sci. 2012;11(1):1–8.
7. Alp M, Midilli M, Kocabağli N, Yilmaz H, Turan N, Gargili A, Acar N. The effects of dietary oregano essential oil on live performance, carcass yield, serum immunoglobulin G level, and oocyst count in broilers. Journal of Applied Poultry Research. 2012;21(3):630–6.
8. Gaafar K, Abou-Elkhair R, Helal MA, Gaafar KM, Abou-Elkhair R, Elbahy NM, Helal MA, Mahboub HDH, Sameh G. Bioactive Effect of Dietary Supplementation with Essential Oils Blend of Oregano, Thyme and Garlic oils on Performance of Broilers Infected with Eimeria species. Glob Vet [Internet]. 2014;13(6):977–85. Available from: https://www.researchgate.net/publication/331998501
9. Bozkurt M, Aysul N, Küçükyilmaz K, Aypak S, Ege G, Çatli AU, Akşit H, Çöven F, Seyrek K, Çinar M. Efficacy of in-feed preparations of an anticoccidial, multienzyme,

prebiotic, probiotic, and herbal essential oil mixture in healthy and Eimeria spp.-infected broilers. Poult Sci. 2014;93(2):389–99.

10. Barbour EK, Bragg RR, Karrouf G, Iyer A, Azhar E, Harakeh S, Kumosani T. Control of eight predominant Eimeria spp: Involved in economic coccidiosis of broiler chicken by a chemically characterized essential oil. J Appl Microbiol. 2015 Mar 1;118(3):583–91.
11. Murakami AE, Eyng C, Torrent J. Effects of functional oils on coccidiosis and apparent metabolizable energy in broiler chickens. Asian-Australas J Anim Sci. 2014;27(7):981–9.
12. Mohiti-Asli M, Ghanaatparast-Rashti M. Dietary oregano essential oil alleviates experimentally induced coccidiosis in broilers. Prev Vet Med. 2015 Jun 15;120(2):195–202.
13. Bozkurt M, Ege G, Aysul N, Akşit H, Tüzün AE, Küçükyllmaz K, Borum AE, Uygun M, Akşit D, Aypak S, Simşek E, Seyrek K, Koçer B, Bintaş E, Orojpour A. Effect of anticoccidial monensin with oregano essential oil on broilers experimentally challenged with mixed Eimeria spp. Poult Sci. 2016 Aug 1;95(8):1858–68.
14. Yang C, Kennes YM, Lepp D, Yin X, Wang Q, Yu H, Yang C, Gong J, Diarra MS. Effects of encapsulated cinnamaldehyde and citral on the performance and cecal microbiota of broilers vaccinated or not vaccinated against coccidiosis. Poult Sci. 2020 Feb 1;99(2):936–48.
15. Upadhaya SD, Cho SH, Chung TK, Kim IH. Anti-coccidial effect of essential oil blends and vitamin D on broiler chickens vaccinated with purified mixture of coccidian oocyst from Eimeria tenella and Eimeria maxima. Poult Sci. 2019 Jul 1;98(7):2919–26.
16. Lee JW, Kim DH, Kim YB, Jeong SB, Oh ST, Cho SY, Lee KW. Dietary encapsulated essential oils improve production performance of coccidiosis-vaccine-challenged broiler chickens. Animals. 2020 Mar 1;10(3).
17. Hafeez A, Ullah Z, Khan RU, Ullah Q, Naz S. Effect of diet supplemented with coconut essential oil on performance and villus histomorphology in broiler exposed to avian coccidiosis. Trop Anim Health Prod. 2020 Sep 1;52(5):2499–504.
18. Gordillo Jaramillo FX, Kim DH, Lee SH, Kwon SK, Jha R, Lee KW. Role of oregano and Citrus species-based essential oil preparation for the control of coccidiosis in broiler chickens. J Anim Sci Biotechnol. 2021 Dec 1;12(1).
19. Langerudi MT, Youssefi MR, Tabari MA. Ameliorative effect of Psidium guajava essential oil supplemented feed on chicken experimental coccidiosis. Trop Anim Health Prod. 2022 Mar 1;54(2).

20. Zhang L, Wang X, Huang S, Huang Y, Shi H, Bai X. Effects of dietary essential oil supplementation on growth performance, carcass yield, meat quality, and intestinal tight junctions of broilers with or without Eimeria challenge. Poult Sci. 2023 Sep 1;102(9).
21. Youssefi MR, Alipour R, Fakouri Z, Shahavi MH, Nasrabadi NT, Tabari MA, Crescenzo G, Zizzadoro C, Centoducati G. Dietary Supplementation with Eugenol Nanoemulsion Alleviates the Negative Effects of Experimental Coccidiosis on Broiler Chicken's Health and Growth Performance. Molecules. 2023 Mar 1;28(5).
22. Fritzlen CJ, Wilson KM, Samper JM, Persia ME. Effects of essential oils and betaine on male broilers raised on used litter seeded with coccidia oocysts. Journal of Applied Poultry Research. 2024 Jun 1;33(2).
23. Khukhodziinai JS, Das PK, Mukherjee J, Banerjee D, Ghosh PR, Das AK, Samanta I, Jas R, Mondal S, Patra AK. Effect of Dietary Benzoic Acid and Oregano Essential Oil as a Substitute for an Anti-Coccidial Agent on Growth Performance and Physiological and Immunological Responses in Broiler Chickens Challenged with Eimeria Species. Animals. 2024 Oct 1;14(20).
24. Elbaz AM, Ashmawy ES, Mourad DM, Amin SA, Khalfallah EKM, Mohamed ZS. Effect of oregano essential oils and probiotics supplementation on growth performance, immunity, antioxidant status, intestinal microbiota, and gene expression in broilers experimentally infected with Eimeria. Livest Sci. 2025 Jan 1;291.
